# Supraventricular tachyarrhythmias in arrhythmogenic cardiomyopathy and Brugada syndrome: a systematic review

**DOI:** 10.3389/fcvm.2026.1876080

**Published:** 2026-07-30

**Authors:** Luca Canovi, Laura Rotondo, Francesco Vitali, Martina De Raffele, Angelo Melpignano, Cristina Balla, Michele Malagù, Luigi Pannone, Carlo de Asmundis, Matteo Bertini, Elisabetta Tonet

**Affiliations:** 1 Cardiovascular Institute, Azienda Ospedaliero-Universitaria of Ferrara, Ferrara, Italy; 2 Heart Rhythm Management Centre, Postgraduate Program in Cardiac Electrophysiology and Pacing, Universitair Ziekenhuis Brussel–Vrije Universiteit Brussel, European Reference Networks Guard-Heart, Brussels, Belgium

**Keywords:** arrhythmogenic cardiomyopathy, atrial fibrillation, atrial flutter, atrial substrate, Brugada syndrome, sudden cardiac death, supraventricular tachyarrhythmias

## Abstract

**Background:**

Arrhythmogenic cardiomyopathy (ACM) and Brugada syndrome (BrS) are inherited arrhythmogenic disorders historically defined by their predisposition to ventricular arrhythmias and sudden cardiac death (SCD). Accumulating evidence indicates that both conditions also involve the atrial myocardium and are associated with a high prevalence of supraventricular tachyarrhythmias (SVT). A clearer understanding of their mechanisms and clinical impact is essential to optimize risk stratification and management.

**Methods:**

A systematic review was conducted in accordance with the PRISMA 2020 statement. PubMed and the Cochrane Library were searched up to December 2025 for studies reporting atrial fibrillation (AF), atrial flutter (AFL), atrioventricular nodal re-entrant tachycardia (AVNRT), or atrioventricular re-entrant tachycardia (AVRT) in patients with ACM or BrS. Of 487 records identified, 486 were screened after removal of one duplicate, and 30 studies met the inclusion criteria. Data on atrial substrate, SVT prevalence, mechanisms, and clinical outcomes were extracted.

**Results:**

SVT were consistently more frequent in both ACM and BrS than in age-matched general populations. AF was the most common arrhythmia, often arising at a young age and in structurally normal atria. Across studies, SVT were linked to inappropriate implantable cardioverter-defibrillator (ICD) shocks, heart failure progression, recurrence of ventricular arrhythmias, and an elevated thromboembolic risk that was frequently underestimated by the CHA₂DS₂-VASc score.

**Conclusions:**

SVT represent a clinically relevant manifestation of ACM and BrS and carry prognostic implications for arrhythmic monitoring, ICD programming, and thromboembolic risk assessment. Prospective studies are needed to refine risk-stratification tools and to clarify the mechanistic overlap between these inherited arrhythmogenic syndromes.

## Graphical Abstract

**Figure:**
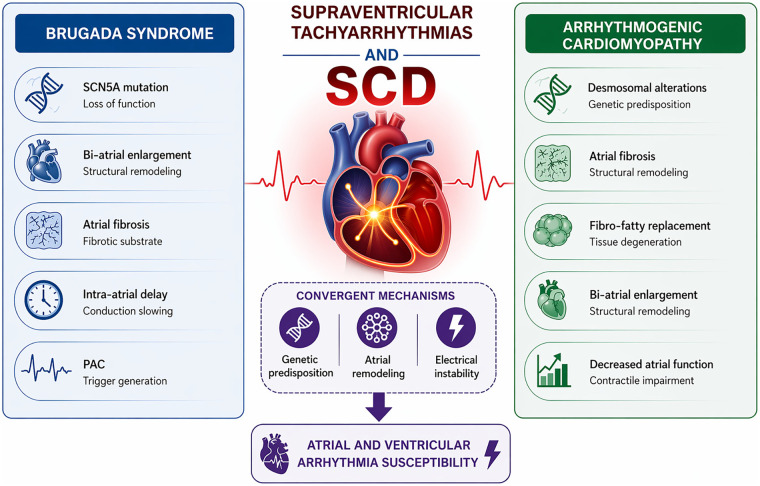
Overview of the shared atrial and ventricular phenotypes of Brugada syndrome and arrhythmogenic cardiomyopathy, including their respective substrates for supraventricular tachyarrhythmias and their convergence toward sudden cardiac death (SCD). PAC, premature atrial contractions.

## 1 Introduction

Arrhythmogenic cardiomyopathy (ACM) and Brugada syndrome (BrS) are inherited, genetically determined disorders characterized by an increased risk of ventricular arrhythmias (VA) and sudden cardiac death (SCD). ACM, caused by pathogenic variants in desmosomal proteins, induces structural myocardial alterations that progress from the epicardium to the endocardium (1). Depending on the specific desmosomal involvement, the disease may predominantly affect the right, the left, or both ventricles. In contrast, BrS results from variants affecting cardiac ion-channel proteins (2); the resulting ionic-current abnormalities mirror the regional expression of the affected channels and are most pronounced in the epicardial layer of the right ventricular outflow tract (RVOT) (3, 4).

On the basis of overlapping phenotypic and pathophysiological features, a unifying hypothesis has been proposed in which ACM and BrS represent different expressions of a shared disease spectrum (5, 6). Historically, clinical interest has focused on ventricular arrhythmias and SCD risk in both conditions. More recently, attention has shifted to atrial involvement, which is present in both diseases, although to varying degrees. These observations delineate a spectrum of atrioventricular involvement and underscore the need to investigate the underlying mechanisms in both atrial and ventricular myocardium.

The aim of the present systematic review is to summarize the current evidence on atrial substrate and on the prevalence, mechanisms, and prognostic implications of supraventricular tachyarrhythmias (SVT) in ACM and BrS, and to outline the clinical implications for risk stratification and management.

## 2 Methods

### 2.1 Study design and protocol

This systematic review was designed and reported in accordance with the Preferred Reporting Items for Systematic Reviews and Meta-Analyses (PRISMA) 2020 statement. A formal review protocol was developed *a priori* by the investigators; the review was not prospectively registered in PROSPERO. The completed PRISMA 2020 checklist is provided as Supplementary Table S1.

### 2.2 Eligibility criteria

The research question was structured according to the PICOS framework, adapted for observational studies of prevalence and clinical association. Participants—adult patients with a confirmed diagnosis of ACM (per Task Force Criteria 1994/2010 or Padua Criteria 2020) or BrS (spontaneous or drug-induced type 1 ECG pattern). Index condition—the inherited arrhythmogenic syndrome itself; no therapeutic intervention was studied, since the underlying disease was the exposure of interest. Comparators—when reported, age-matched general-population reference data, healthy controls, or within-cohort comparisons (e.g., genotype subgroups, presence vs. absence of SVT). Outcomes—the primary outcome was the prevalence of AF, AFL, AVNRT, AVRT, or pooled SVT; secondary outcomes were clinical correlates and prognostic implications, including ICD shocks, heart failure, thromboembolic events, ventricular arrhythmia recurrence, and mortality. Study designs—observational studies (cross-sectional, case-control, retrospective and prospective cohort) and randomized clinical trials.

Reviews, editorials, case reports, case series, and non-human studies were excluded. Studies reporting aggregated data on SVT as a single group were considered eligible provided that the group comprised only the four arrhythmias above. No language restriction was applied.

### 2.3 Information sources and search strategy

PubMed (via the National Center for Biotechnology Information interface) and the Cochrane Library were systematically searched from inception up to December 2025 (date of last search: 31 December 2025). The following PubMed search string was used for ACM:

(((“arrhythmogenic cardiomyopathy”[MeSH Terms]) OR (ACM) OR (ARVC) OR (ARVD) OR (right ventricular dysplasia) OR (arrhythmogenic right ventricular cardiomyopathy)) AND ((“supraventricular arrhythmia”[MeSH Terms]) OR (supraventricular) OR (atrial fibrillation) OR (AF) OR (atrial flutter) OR (supraventricular tachycardia) OR (SVT) OR (supraventricular tachyarrhythmia) OR (SVTA))).

The following PubMed search string was used for BrS:

(((“Brugada syndrome”[MeSH Terms]) OR (Brugada) OR (BrS)) AND ((“supraventricular arrhythmia”[MeSH Terms]) OR (supraventricular) OR (atrial fibrillation) OR (AF) OR (atrial flutter) OR (supraventricular tachycardia) OR (SVT) OR (supraventricular tachyarrhythmia) OR (SVTA))).

The same conceptual search strategy was translated and applied to the Cochrane Library. In addition, the reference lists of all retrieved articles were hand-searched to identify further eligible studies.

### 2.4 Study selection and data extraction

Records were screened by title and abstract by two independent reviewers, with disagreements resolved by consensus or by a third reviewer. Full-text articles of potentially eligible records were retrieved and assessed against the eligibility criteria. For each included study, the following data were extracted: first author, publication year, study design, sample size, mean or median age, diagnostic criteria for ACM (Task Force Criteria 1994/2010 or Padua Criteria 2020) and for BrS (spontaneous vs. drug-induced type 1 pattern), monitoring strategy (routine ECG/Holter vs. cardiac implantable electronic devices), prevalence of any SVT and of each subtype, clinical correlates, and reported outcomes.

### 2.5 Synthesis approach

Owing to the methodological heterogeneity of the included studies—which differed in design, cohort composition, diagnostic criteria, and monitoring strategy—and to the prevalence-based nature of the extracted outcomes, a quantitative meta-analysis was not performed. Findings were synthesized qualitatively and stratified by underlying disease (ACM or BrS) and by SVT subtype.

### 2.6 Risk of bias and quality assessment

Methodological quality of the included studies was appraised narratively at the study-set level rather than by formal item-level scoring of individual papers, given that all included studies addressed prevalence and clinical association rather than treatment effects, were derived from heterogeneous observational designs, and reported predominantly aggregate descriptive measures. Four critical-appraisal domains derived from the Joanna Briggs Institute checklist for studies reporting prevalence data and from the Newcastle–Ottawa Scale were used as a framework: (i) representativeness of the study population, (ii) appropriateness of the arrhythmia ascertainment method, (iii) consistency of outcome definitions, and (iv) follow-up duration. The corresponding domain-level appraisal of the included study set is reported in Section 3.2 of the Results, and methodological heterogeneity is further addressed in the Limitations subsection of the Discussion.

## 3 Results

### 3.1 Study selection

**Figure 1:**
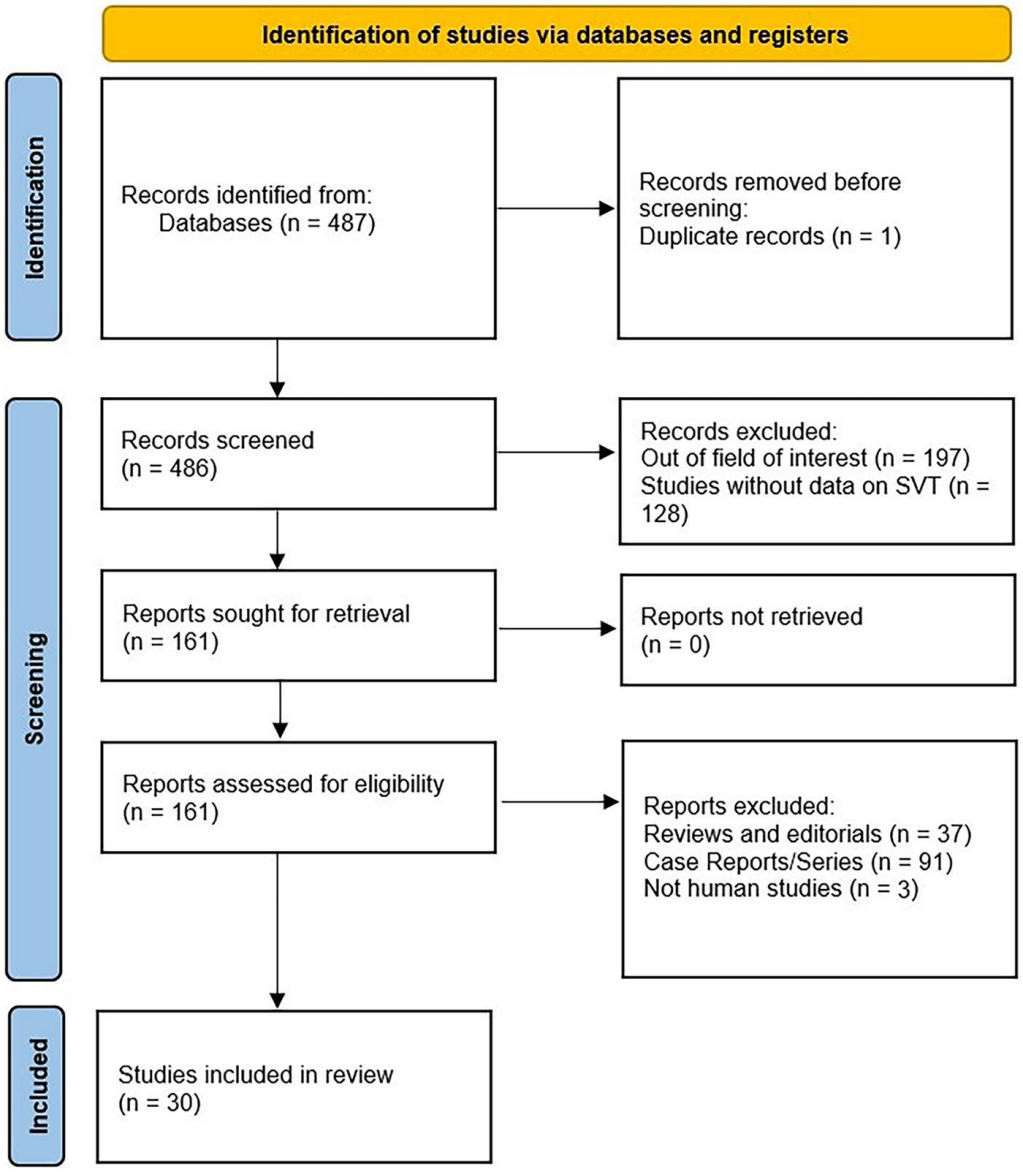
PRISMA 2020 flow diagram of study selection. Flow diagram summarizing the identification, screening, eligibility assessment, and inclusion of studies, in accordance with the PRISMA 2020 guidelines.

The literature search yielded 487 records, reduced to 486 after removal of one duplicate. Title and abstract screening excluded 325 records (197 outside the field of interest and 128 without data on SVT). Of the 161 reports assessed for eligibility, 131 were excluded (37 reviews and editorials, 91 case reports or case series, and 3 non-human studies). Thirty studies were finally included in the qualitative synthesis (Figure 1).

### 3.2 Critical appraisal of included studies

Most included studies were retrospective observational analyses with intermediate sample sizes, ranging from 21 to 560 patients. Population representativeness was a recognized concern in two opposite directions: proband-based ICD cohorts [e.g., Sacher et al. (7), Sarkozy et al. (8), Schimpf et al. (9), De Asmundis et al. (10)] may have overrepresented patients with severe phenotypes, whereas family-based or population-screening cohorts may have underestimated symptomatic SVT, and pediatric or genotype-restricted subgroups [e.g., Bourfiss et al. (11)] are not generalizable to the overall ACM/BrS population.

Arrhythmia ascertainment differed substantially across studies and likely accounts for a large fraction of the observed between-study heterogeneity in SVT prevalence. Studies based on continuous device monitoring with implantable cardioverter-defibrillators or implantable loop recorders [e.g., Bergonti et al. (12), BruLoop study; Chu et al. (13); Sarkozy et al. (8)] systematically captured higher SVT rates than studies relying on routine clinic visits or symptom-triggered ECG/Holter assessment [e.g., Babai Bigi et al. (14), Tonet et al. (15)]. Outcome definitions were consistent for AF and AFL but more heterogeneous for AVNRT, AVRT, and pooled “other SVT” categories, with several studies aggregating these subtypes without further specification.

Follow-up duration was variable, ranging from cross-sectional snapshots to multi-year longitudinal observation [median follow-up 33.7 months in the BruLoop study, up to 48 ± 38 months in the Calò et al. (16) cohort]. Risk of selection bias was unevenly distributed across the study set: highest in single-center, ICD-only series, and lowest in multicenter registry-based cohorts (e.g., ESC EORP Cardiomyopathy Registry, Nordic ARVC Registry). Reporting bias toward positive associations between SVT and ventricular arrhythmias or adverse outcomes cannot be excluded. Despite these limitations, the consistency of the principal findings across heterogeneous designs, settings, and monitoring strategies supports the qualitative validity of the synthesis. The implications of this methodological heterogeneity are further considered in the Limitations subsection of the Discussion.

### 3.3 Atrial substrate in Brugada syndrome

Electrical abnormalities in BrS predominantly affect the epicardial region of the RVOT, where the mutated ion channels are most highly expressed. However, as a genetic disorder, BrS entails widespread myocardial alterations of variable intensity (17). It is therefore plausible that primary ion-channel dysfunction also involves atrial myocytes, given the known expression of *SCN5A* in atrial tissue (18). This impairs atrial electrical function, increases atrial vulnerability, and predisposes to supraventricular arrhythmias.

From a pathophysiological perspective, pathogenic *SCN5A* variants delay the recovery of the sodium channel from the inactive state and prolong the monophasic action potential duration (MAPD) in cardiomyocytes (19). A hallmark of BrS is the regional heterogeneity of MAPD, due to variable ion-channel expression and dysfunction across different myocardial areas. Choi et al. (20) demonstrated that, although absolute MAPD does not differ significantly between patients with BrS and controls, MAPD dispersion is significantly greater in BrS. This increased repolarization dispersion in the atria reflects the zonal electrical heterogeneity that predisposes to supraventricular tachyarrhythmias.

Consistent with these findings, atrial conduction time is prolonged as a consequence of sodium-channel dysfunction. Bisignani et al. (21) showed that BrS patients harbouring pathogenic *SCN5A* variants exhibited longer atrial conduction times—comparable to those observed after class Ic antiarrhythmic drug administration in *SCN5A*-negative patients. Notably, prolonged atrial conduction time correlated with the occurrence of AF, and BrS patients with AF had non-dilated left atria, further underscoring the primarily electrical nature of AF in this setting.

Morita et al. (22) described increased atrial fibrosis in BrS, evidenced by fragmented atrial electrograms and intra-atrial conduction delay during electrophysiological study. These abnormalities, together with frequent repetitive atrial firing, create an arrhythmic substrate favoring both focal and re-entrant atrial tachyarrhythmias. In the same cohort, BrS patients exhibited prolonged AH and HV intervals and an extended atrioventricular nodal effective refractory period, which may predispose to AVNRT in the presence of dual nodal physiology.

Growing evidence also supports structural atrial remodelling in BrS. Toh et al. (23) reported a significant increase in left atrial size in BrS patients compared with healthy controls; importantly, these subjects showed no evidence of diastolic dysfunction, suggesting that left atrial enlargement was attributable to primary myocardial damage. Diomede et al. (24) reported significant right atrial enlargement together with impaired biatrial longitudinal and circumferential strain. Taken together, these findings indicate that atrial electroanatomical remodelling is an integral component of BrS pathophysiology.

Finally, Babai Bigi et al. (14) identified a spontaneous type 1 Brugada ECG pattern and a history of life-threatening cardiac events as the main risk factors for AF in BrS. This finding reinforces the link between the severity of cardiac ion-channel dysfunction and susceptibility to both atrial and ventricular arrhythmias. In this context, increased vagal tone—known to trigger ion-channel dysfunction and ventricular fibrillation in BrS—may also contribute to AF onset. The main studies addressing SVT in BrS are summarized in Table 1.

**Table 1:** Studies reporting supraventricular tachyarrhythmias in Brugada syndrome.

References	*N* pts	SVT % (*n*)	AF % (*n*)	AFL % (*n*)	Other SVT % (*n*)	Mean age, year
Sacher et al. (7)	220	15 (32)	72 (23)	9 (3)	19 (6)	46
Babai Bigi et al. (14)	115	–	13 (15)	–	–	29
Takagi et al. (25)	188	–	17 (32)	–	–	–
Sarkozy et al. (8)	47	–	11 (5)	–	–	44.5
Kusano et al. (26)	73	–	13.7 (10)	–	–	53
Schimpf et al. (9)	115	23 (26)	50 (13)^a^	–	50 (13)^b^	45
Tokioka et al. (27)	246	–	17.9 (44)	–	–	–
Giustetto et al. (28)	560	9 (48)^c^	–	–	–	47
Calò et al. (16)	347	–	5.2 (18)	–	–	–
De Asmundis et al. (10)	289	28.4 (82)	65 (53)	21 (17)	28 (23)	45
Tse et al. (29)	275	–	5 (14)	–	–	53
Bergonti et al. (12)	370	15.7 (58)	69 (40)	–	33 (19)	43.5

AF, atrial fibrillation; AFL, atrial flutter; SVT, supraventricular tachyarrhythmia(s). “Other SVT” includes AVNRT, AVRT, focal atrial tachycardia, and junctional tachycardia. Percentages in the AF, AFL and “Other SVT” columns refer to patients with documented SVT in the cited study; absolute numbers are reported in parentheses. Dashes indicate data not reported or not applicable.

a Reported as combined AF/AFL in the original paper.

b Includes 8 AVNRT, 2 AVRT and 3 atrial tachycardias.

c Combined AF and AFL.

### 3.4 Atrial substrate in arrhythmogenic cardiomyopathy

Although ACM predominantly affects the ventricles, atrial alterations and a predisposition to SVT are increasingly recognized as part of the clinical spectrum of the disease. Across published series, the reported prevalence of SVT in ACM ranges from 14% to 42%. This variability is likely attributable to differences in cohort composition. The highest prevalence was reported by Chu et al. (13) in a cohort of patients referred for catheter ablation of drug-refractory ventricular arrhythmias, nearly all (97%) of whom carried an implantable cardioverter-defibrillator (ICD). Lower prevalence rates have generally been reported in the absence of continuous ICD monitoring or when earlier-stage patients were included (30).

The prevalence of SVT in ACM is considerably higher than in the age-matched general population, with an earlier age at onset. For instance, in the series reported by Saguner et al. (31), the mean age was 50 years, and SVT prevalence was 20%, compared with an expected prevalence of approximately 0.5% in the general population at the same age.

Understanding the atrial substrate in ACM is clinically relevant, as several studies have linked SVT to increased morbidity, including life-threatening ventricular arrhythmias, inappropriate ICD shocks, heart failure, heart transplantation, and cardiovascular mortality (13, 30, 31). However, the underlying pathophysiological mechanisms remain incompletely understood. Multiple studies have sought to define the association between SVT and clinical characteristics, identifying significant correlations with right ventricular dilatation, right and left atrial dilatation, and moderate-to-severe tricuspid regurgitation (13, 30, 31–33). These observations suggest that atrial involvement may, at least in part, be a consequence of ventricular disease progression.

Atrial morphological and functional abnormalities have also been documented on imaging (13, 30, 33). Compared with controls, patients with ACM were found to have enlarged atria with reduced function on functional cardiac magnetic resonance (CMR) and impaired right atrial (RA) deformation—even in the presence of a normal RA volume—on speckle-tracking echocardiography (STE)-derived RA strain. Bourfiss et al. (11) showed that ACM patients with *PLN* variants or with no identified variant had increased biatrial volume, whereas patients with *PKP2* mutations displayed smaller atria. Despite the reduced atrial size, SVT prevalence in *PKP2* carriers was comparable to that observed in ACM patients without identified variants, suggesting a different pathophysiological mechanism underlying atrial disease. This finding may reflect the presence of atrial fibrosis in desmosomal variant carriers, possibly preceding overt atrial dilatation; however, atrial fibrosis was not detectable by conventional CMR in that study.

In a subset of patients, SVT onset may precede the occurrence of ventricular arrhythmias, as reported by Brembilla-Perrot et al., who demonstrated an increased inducibility of SVT at electrophysiological study compared with controls, independent of disease stage. These observations support the possibility of primary atrial involvement (34). It therefore appears plausible that the atrial substrate in ACM may result either from primary atrial involvement due to the underlying genetic defect or, more commonly, from the progression of ventricular disease—or from a combination of both mechanisms. The main studies addressing SVT in ACM are summarized in Table 2.

**Table 2:** Studies reporting supraventricular tachyarrhythmias in arrhythmogenic cardiomyopathy.

References	*N* pts	SVT % (*n*)	AF % (*n*)	AFL % (*n*)	Other SVT % (*n*)	Mean age, year
Tonet et al. (15)	72	24 (17)	59 (10)	30 (5)	41 (7)	38
Brembilla-Perrot et al. (34)	47	17 (8)	63 (5)	13 (1)	25 (2)	44
Chu et al. (13)^a^	36	42 (15)	73 (11)	73 (11)	–	52
Camm et al. (30)	248	14 (35)	80 (28)	31 (11)	23 (8)	49
Saguner et al. (31)	90	20 (18)	88 (16)	12 (2)	–	50
Brun et al. (35)	39 fam.	29	–	–	–	–
Wu et al. (36)	294	15 (44)	57 (25)	43 (19)	–	43
Bourfiss et al. (11)	71	27 (19)	89 (17)	16 (3)	–	54
Müssigbrodt et al. (37)	70	37 (26)	65 (17)	23 (6)	42 (11)	59
Cardona-Guarache et al. (32)	117	22 (26)	73 (19)	35 (9)	31 (8)	60
Baturova et al. (38)	301	–	14 (41)	–	–	41
Butters et al. (43)	21	–	14 (3)	–	–	–
Gimeno et al. (39)	128	–	16 (21)	–	–	–
Toso et al. (33)	111	20.7 (23)	65 (15)	34.8 (8)	–	–
Zado et al. (44)	119	34 (40)	77 (31)	67 (27)	25 (10)	–

AF, atrial fibrillation; AFL, atrial flutter; SVT, supraventricular tachyarrhythmia(s). “Other SVT” includes AVNRT, AVRT and focal atrial tachycardia. Percentages in the AF, AFL and “Other SVT” columns refer to patients with documented SVT in the cited study; absolute numbers are reported in parentheses. Dashes indicate data not reported or not applicable.

a In the Chu et al. (10) series, AF and AFL coexisted in 7 of 15 SVT patients, so the AF and AFL columns are not mutually exclusive.

### 3.5 Atrial fibrillation

In 1991, Tonet et al. (15) were the first to investigate the prevalence of SVT in patients with ACM, reporting AF as the most frequent arrhythmia with a prevalence of 13.9%. Notably, all AF episodes occurred in patients already receiving antiarrhythmic therapy for ventricular arrhythmias, yet AF still developed in a substantial proportion of cases. Several subsequent studies confirmed that AF prevalence in ACM is significantly higher than in the general population. Recent estimates range between 8.5% and 30.6%, depending on mean age and monitoring strategy (e.g., routine visits or symptom-triggered assessment vs. home-monitored ICDs) (13, 30, 32, 36). These figures are markedly higher than the 5.9% reported in the general population in the current European Society of Cardiology (ESC) AF guidelines (40).

Beyond its increased prevalence, AF in ACM is notable for its early onset. Baturova et al. (38) reported a 14% AF prevalence in a relatively young cohort (median age 41 years), compared with an expected prevalence of only 1%–2% in the age-matched general population. Camm et al. (30) confirmed a 14% prevalence with a mean age at AF diagnosis of 43 ± 14 years, consistent with an unusually early onset. These findings are in line with data from the ESC EORP Cardiomyopathy Registry, which reported an AF prevalence of 16% among 128 ACM patients (39).

Genetic background appears to play a key role in AF predisposition. Brun et al. (35) analyzed 39 ACM families and stratified them according to desmosomal gene mutations (*DSP, PKP2, DSG2, DSC2*), *TTN* mutations, or absence of identified variants. The *TTN* group showed the highest prevalence of supraventricular arrhythmias (46% vs. 13%; *p* = 0.013). Beyond its prevalence, AF in ACM carries substantial clinical implications. Müssigbrodt et al. (37) analyzed 70 ACM patients and reported an AF prevalence of 24.3%. Binary logistic regression identified heart failure (HF) as significantly associated with SVT (odds ratio (OR) 10.89; 95% CI 1.08–109.96; *p* = 0.043). In a population with widespread ICD use, SVT further increased the risk of inappropriate shocks, which are themselves associated with HF progression and mortality. In patients with prior ventricular tachycardia (VT) ablation, Cox regression identified SVT as an independent predictor of VT recurrence (hazard ratio (HR) 3.01; 95% CI 1.15–7.89; *p* = 0.025), suggesting a potential role for SVT in arrhythmic risk stratification.

A further clinically relevant observation was provided by Toso et al. (33), who showed that the cardioembolic risk in ACM patients is substantially higher than in the general population (HR 4.12) and occurs at a younger age (mean 42 ± 9 years vs. >60 years). The CHA₂DS₂-VASc score failed to predict cardioembolic events, as 60% of affected patients had a pre-event score of 0 (or 1 if female)—typically associated with low embolic risk (0.9%–1.3% per year). Moreover, only 45.5% of cardioembolic events were temporally associated with SVT, supporting the hypothesis that atrial myopathy *per se* contributes to embolic risk independently of documented SVT. Of note, the 2024 ESC AF guidelines have simplified the score to CHA₂DS₂-VA, removing the female-sex modifier; nevertheless, all studies cited herein applied the prior CHA₂DS₂-VASc formulation (16).

Finally, Saguner et al. (31) compared 90 ACM patients with and without AF/AFL and identified right ventricular dysfunction—defined as fractional area change <27%—as a significant risk factor for AF/AFL. This observation suggests that right ventricular dysfunction, a hallmark of ACM progression, may affect both ventricular and supraventricular arrhythmogenesis. AF/AFL was also more frequent in patients with inappropriate ICD shocks (36% vs. 9%; *p* = 0.03), a finding of practical relevance when considering single- vs. dual-chamber ICD implantation. Importantly, AF/AFL was significantly associated with heart transplantation and cardiovascular mortality (56% vs. 16%; *p* = 0.014), underscoring its prognostic value in ACM.

In BrS, AF prevalence is likewise significantly higher than in the general population. In an early multicenter study, Sacher et al. (7) analyzed 220 BrS patients who had undergone ICD implantation (mean age 46 years) and reported an SVT prevalence of 15%, with AF being the most common arrhythmia (10.5% of the overall cohort). In 2007, Sarkozy et al. (8) retrospectively analyzed 47 BrS patients who had undergone primary-prophylactic ICD implantation, reporting an AF prevalence of 11%—substantially higher than in age-matched controls (mean age 44.5 ± 15 years). AF was also an independent predictor of shorter inappropriate-shock-free survival (*p* = 0.005). These findings were later corroborated by Bergonti et al. (12) in the BruLoop study, which enrolled 370 BrS patients (mean age 43.5 ± 15.9 years) with implantable loop recorders: over a median follow-up of 33.7 months, AF episodes were recorded in 40 patients (10.8%).

A higher prevalence was reported by De Asmundis et al. (10) in 289 BrS probands (mean age 45 ± 16 years), in whom AF prevalence reached 18.3%; in 35.8% of cases AF was the first clinical manifestation of a previously unrecognized BrS. Notably, despite a low mean CHA₂DS₂-VASc score, the incidence of cerebrovascular events was unexpectedly high (15.1%), suggesting—as in ACM—that cardioembolic risk in BrS may be underestimated by conventional risk-stratification tools.

A small number of studies have explored the relationship between atrial and ventricular arrhythmias in BrS. In 2007, Takagi et al. (25) prospectively analyzed 188 BrS patients (AF prevalence 17%) and found that a history of AF was significantly more frequent in patients with ventricular fibrillation (VF) or syncope than in asymptomatic individuals (*p* = 0.04). Similarly, Kusano et al. (26) analyzed 73 BrS patients (AF prevalence 13.7%) and found that spontaneous AF was associated with a higher incidence of syncope (60.0% vs. 22.2%; *p* = 0.03) and documented VF (40.0% vs. 14.3%; *p* = 0.05). More recently, Tokioka et al. (27) retrospectively analyzed 246 BrS patients (13 with a VF history and 40 with syncope): AF prevalence was 17.9% and occurred more frequently in patients with VF/SCD than in those without. Consistent with these findings, Calò et al. (16) analyzed 347 BrS patients (AF prevalence 5.2%), none of whom had a history of cardiac arrest; during a 48 ± 38-month follow-up, 9.2% experienced cardiac arrest, and multivariable analysis confirmed AF as an independent predictor of VF/SCD (HR 3.7; 95% CI 1.59–8.73; *p* = 0.0024).

Overall, these data indicate that—particularly in patients with structurally normal atria—AF prevalence is significantly higher in BrS than in age-matched controls, and that AF and the underlying atrial substrate may represent markers of increased risk for VF and SCD. These observations reinforce the need for careful arrhythmic risk assessment in BrS.

### 3.6 Atrial flutter

Data on AFL prevalence in ACM are limited, mainly because of the historical focus on ventricular arrhythmias and because SVT have often been analyzed collectively or distinguished only from AF. As for AF, Tonet et al. (15) were the first to investigate AFL prevalence in ACM in 1991: in 72 consecutive patients (mean age 38 years) referred for ventricular arrhythmias, AFL prevalence was 6.9%. Two additional studies support these findings. In a cross-sectional study of 294 consecutive patients with ACM (reported as ARVC in the original study), Wu et al. (36) reported an AFL prevalence of 6.5%. Similarly, Cardona-Guarache et al. retrospectively assessed 117 patients with ACM and reported an AFL prevalence of 7.7% (32).

Few studies have specifically addressed the clinical impact of AFL in ACM. Müssigbrodt et al. (37) reported an AFL prevalence of 8.6%, in line with previous data, and identified HF as significantly associated with SVT on binary logistic regression (OR 10.89; 95% CI 1.08–109.96; *p* = 0.043). Chu et al. (13), in a small cohort of 36 ACM patients—nearly all (97%) carrying an ICD for secondary prevention—identified 11 patients with documented AFL. This remarkably high prevalence likely reflects the advanced disease stage of the cohort and the enhanced diagnostic capability provided by cardiac implantable electronic devices (CIEDs). Clinically, 8.3% of these patients experienced inappropriate ICD shocks attributable to SVT. Similarly, Camm et al. (30) analyzed 248 ACM patients from a large registry and observed that SVT occurred at a younger age than in the general population and were clinically meaningful, being associated with inappropriate ICD shocks, HF, and mortality.

**Figure 2:**
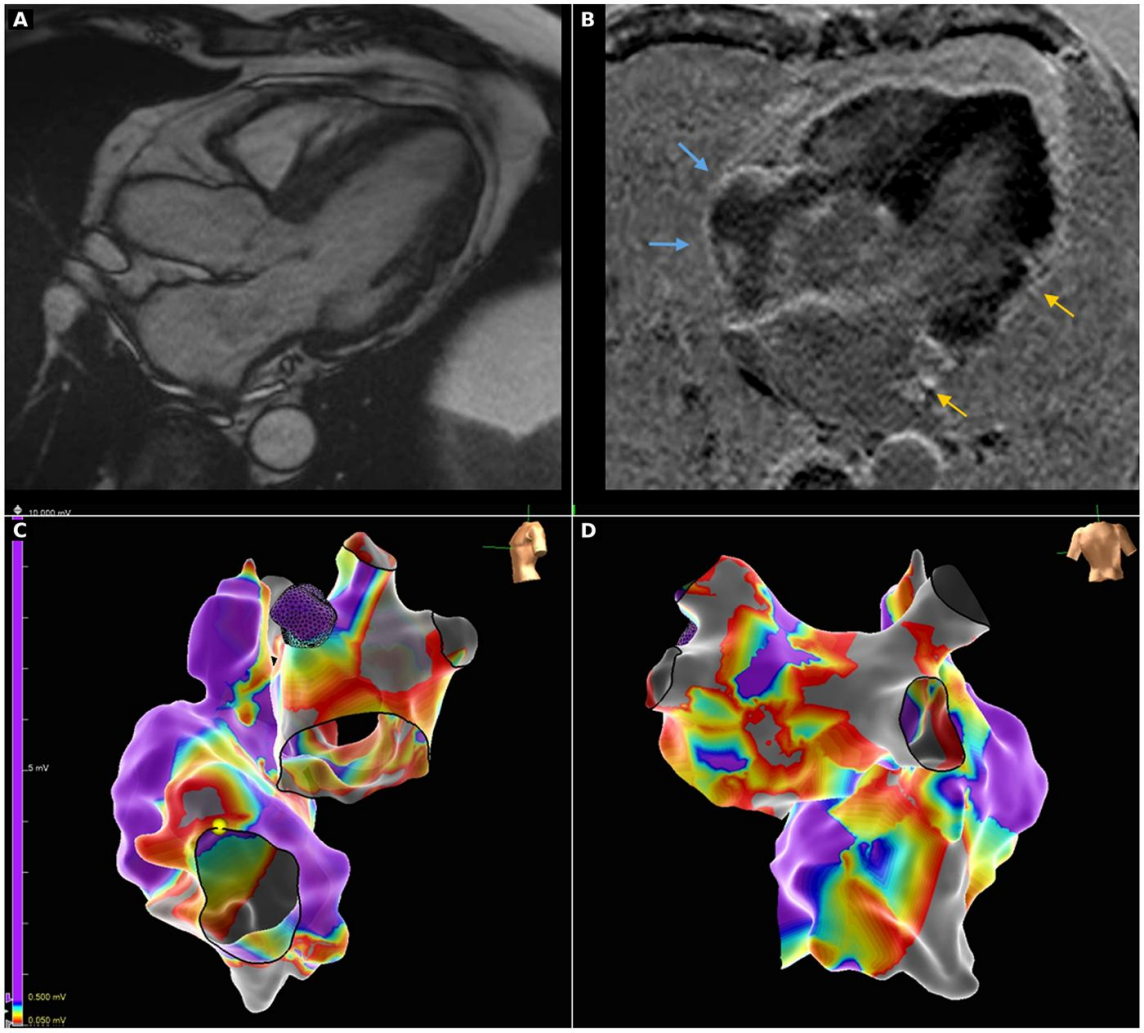
Multimodal imaging and electroanatomical mapping in arrhythmogenic cardiomyopathy (ACM). **(A)** Cine three-chamber CMR image showing left-ventricular involvement with fatty infiltration of the infero-lateral wall (yellow arrow). **(B)** Late gadolinium enhancement (LGE) four-chamber view showing fibrosis of the left-ventricular lateral wall (yellow arrows) and of both atria (blue arrows). **(C,D)** Biatrial voltage maps obtained with the EnSite X system (Abbott), shown in two complementary projections. Extensive low-voltage areas are evident in both atria, consistent with diffuse biatrial fibrosis and defining a complex arrhythmogenic substrate that predisposes to supraventricular tachyarrhythmias. All panels are original and were acquired at the authors’ institutions; the figure has not been published previously.

Although data are limited, AFL appears to be more prevalent in ACM patients than in the age-matched general population (reported rates 6.5%–8.6%) and to be associated with a disproportionately increased risk of cardioembolic events, inappropriate ICD shocks, and HF. Further studies are needed to confirm these findings. An example of biatrial fibrosis and abnormal voltage mapping in an ACM patient with atrial flutter is shown in Figure 2.

In BrS, data on AFL are similarly limited, as most studies report only the combined prevalence of AF and AFL. Schimpf et al. (9) analyzed a cohort of 115 consecutive BrS patients from three European centers and reported an AF/AFL prevalence of 11.0%. Similarly, Giustetto et al. (28) reported an overall prevalence of approximately 9% in the Brugada Registry of the Piedmont region. However, careful assessment of the populations analyzed is essential. To accurately estimate AFL prevalence in BrS, only patients with a spontaneous type 1 Brugada pattern should be considered: otherwise, individuals initially diagnosed with AF in whom a Brugada ECG pattern is subsequently unmasked by sodium-channel blocker therapy may distort the data, since these drugs can both organize AF into typical AFL and disclose the Brugada pattern.

**Figure 3:**
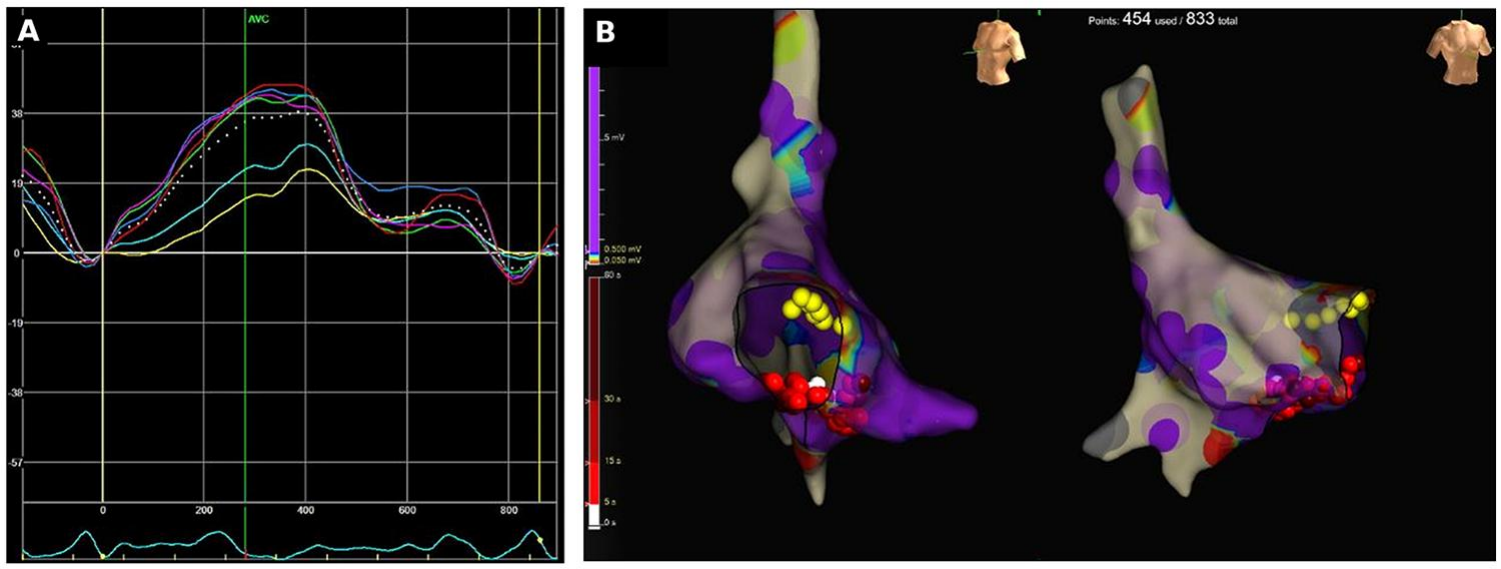
Right atrial functional assessment and electroanatomical mapping during typical atrial flutter ablation. **(A)** Right atrial strain curves showing preserved right atrial deformation. **(B)** Right atrial voltage map obtained with the EnSite X system (Abbott) during a typical atrial flutter ablation procedure (left: left anterior oblique view; right: right anterior oblique view). The voltage map shows a healthy right atrium with preserved voltages. Yellow point tags mark the atrioventricular node and the His-bundle region; red point tags, color-coded according to radiofrequency delivery duration, indicate lesions at the cavotricuspid isthmus. All panels are original and were acquired at the authors’ institutions; the figure has not been published previously.

In this respect, Giustetto et al. (28) reported that patients with a spontaneous Brugada ECG pattern preceding AF/AFL (group 1) were younger than those in whom AF/AFL was the first manifestation and the Brugada ECG was unmasked by class Ic antiarrhythmic drugs (group 2); furthermore, group 1 had a worse prognosis. These findings suggest that patients with a spontaneous Brugada ECG pattern display a distinct pathophysiology compared with those with a drug-induced pattern and should therefore be analyzed separately. Among the few studies specifically assessing AFL prevalence in BrS, Sacher et al. (7) reported a typical AFL prevalence of 1.4% (3/220) in their multicenter ICD cohort, while De Asmundis et al. (10) reported a prevalence of 5.9% for typical AFL in a large cohort of probands. Overall, although data are limited, AFL prevalence in BrS appears significantly higher than in the age-matched general population, most likely reflecting the characteristic atrial substrate of BrS that predisposes to supraventricular arrhythmias (Figure 3).

### 3.7 Atrioventricular nodal re-entrant and atrioventricular re-entrant tachycardias

Specific data on AVNRT and AVRT are scarce, since the studies analyzed focused primarily on AF and AFL or pooled data under the heading of SVT. In ACM, only Müssigbrodt et al. (37) reported a single case of AVNRT, corresponding to 1.4% of the cohort. In BrS, these arrhythmias were mentioned in only two studies: Tse et al. (29) reported one case among 106 patients with BrS presenting with syncope, whereas Schimpf et al. (9) described eight cases of AVNRT and two of AVRT. A proposed mechanism is that prolonged conduction in the perinodal atrial tissue may predispose to AVNRT in patients with dual nodal physiology; however, the precise electrophysiological mechanisms remain to be elucidated. Finally, sporadic reports have described patients with Wolff–Parkinson–White (WPW) syndrome in whom administration of class I antiarrhythmic drugs during electrophysiological study unmasked an underlying BrS (41, 42). The treatment of choice for symptomatic AVNRT or AVRT remains catheter ablation, particularly because these arrhythmias may favor inappropriate ICD interventions.

## 4 Discussion

### 4.1 Summary of main findings

This systematic review of 30 studies confirms that SVT are significantly more prevalent in both ACM and BrS than in the age-matched general population. AF is the most common arrhythmia in these conditions, frequently occurring at an early age and in structurally normal atria. In ACM, SVT correlate with right ventricular dysfunction and atrial dilatation, whereas in BrS they are mainly associated with sodium-channel dysfunction and zonal atrial electrical heterogeneity. The prognostic relevance of SVT in both conditions includes an increased risk of HF progression, inappropriate ICD interventions, embolic events—often inadequately predicted by conventional risk scores (e.g., CHA₂DS₂-VASc/CHA₂DS₂-VA)—and mortality.

### 4.2 Pathophysiological convergence: a shared “connexome” disease spectrum

The available evidence supports overlapping mechanisms between ACM and BrS, consistent with the hypothesis of a “disease of the connexome”, whereby dysfunction of intercellular junctions, gap junctions, and ion channels generates a spectrum of atrial and ventricular disease. Within this framework, ACM and BrS may be regarded not as isolated entities but as phenotypic expressions along a shared continuum (Graphical Abstract). Atrial involvement, although historically overshadowed by the focus on ventricular arrhythmias, represents a clinically meaningful manifestation of this continuum and a marker of disease severity in both conditions.

### 4.3 Clinical implications

The recognition of SVT in ACM and BrS has direct implications for daily clinical practice. First, systematic surveillance for atrial arrhythmias—by means of long-term ECG monitoring or implantable cardiac monitors—should be considered in symptomatic patients and in those with risk markers (e.g., spontaneous type 1 Brugada pattern, right ventricular dysfunction, biatrial enlargement). Second, the choice between single- and dual-chamber ICDs should account for the elevated risk of inappropriate shocks driven by SVT, particularly in advanced disease stages. Third, given that conventional thromboembolic risk scores systematically underestimate stroke risk in these populations, anticoagulation decisions should integrate disease-specific markers of atrial myopathy beyond the CHA₂DS₂-VASc/CHA₂DS₂-VA score. Finally, in symptomatic patients with AVNRT, AVRT, or typical AFL, catheter ablation remains the treatment of choice and may also reduce inappropriate ICD interventions.

### 4.4 Limitations

This systematic review has several limitations. First, the included studies are heterogeneous in design, cohort size, patient selection (proband-based vs. family-based, ICD carriers vs. general cohorts), and arrhythmia-monitoring strategy (routine ECG/Holter vs. implanted devices), which may account for the substantial variability in reported SVT prevalence. Second, a formal item-level risk-of-bias assessment of individual studies was not performed; methodological quality was instead appraised narratively at the study-set level across four critical-appraisal domains (Section 3.2). Third, case reports and case series were excluded, which may have led to underreporting of rare arrhythmic associations such as AVNRT and AVRT. Fourth, publication bias cannot be excluded, particularly for studies reporting negative or neutral findings on atrial involvement. Finally, heterogeneity in diagnostic criteria for ACM across the study period (Task Force Criteria 1994, 2010, Padua Criteria 2020) and variability in the definition of spontaneous vs. drug-induced BrS may have influenced the reported prevalence estimates. The review was not prospectively registered in PROSPERO.

### 4.5 Conclusions and future directions

Although ACM and BrS differ in their clinical course, they share atrial involvement with clinically relevant supraventricular arrhythmias. Beyond highlighting a pathophysiological overlap, the identification of SVT in these conditions has direct clinical implications: SVT contribute to inappropriate ICD therapies, thromboembolic complications, and may signal disease progression. Systematic evaluation and monitoring of SVT should therefore be incorporated into clinical assessment and influence both risk stratification and therapeutic decisions. Prospective studies are needed to clarify the temporal relationship between disease progression and SVT, to refine embolic risk-stratification tools in these patients, and to assess the role of targeted therapies. At the molecular level, further exploration of connexome dysfunction may help to unify the atrial and ventricular manifestations of ACM and BrS, paving the way toward a more integrated understanding of inherited arrhythmogenic syndromes.

## Data Availability

The raw data supporting the conclusions of this article will be made available by the authors, without undue reservation.

## Supplementary material

Supplementary Table S1 *PRISMA 2020* checklist. Completed Preferred Reporting Items for Systematic Reviews and Meta-Analyses (PRISMA) 2020 checklist for this systematic review, indicating the section and page at which each reporting item is addressed.
