# Supplementary material for: Supraventricular tachyarrhythmias in arrhythmogenic cardiomyopathy and Brugada syndrome: a systematic review
Source: Front Cardiovasc Med. 2026 Jul 30;13:1876080. doi: 10.3389/fcvm.2026.1876080 (PMC13467924; doi:10.3389/fcvm.2026.1876080)
Supplement: Supplementary file 1 [file Table1.docx]

**PRISMA 2020 Checklist**

*Systematic review: "Supraventricular Tachyarrhythmias in Arrhythmogenic Cardiomyopathy and Brugada Syndrome"*

*Reference: Page MJ, McKenzie JE, Bossuyt PM, et al. The PRISMA 2020 statement: an updated guideline for reporting systematic reviews. BMJ 2021;372:n71.*

| Section / Topic | Item # | Checklist item | Location reported |
| --- | --- | --- | --- |
| TITLE |  |  |  |
| Title | 1 | Identify the report as a systematic review. | Title page — title explicitly states 'A Systematic Review'. |
| ABSTRACT |  |  |  |
| Abstract | 2 | See the PRISMA 2020 for Abstracts checklist. | Abstract section — structured as Background / Methods / Results / Conclusions; PRISMA-Abstract checklist completed separately. |
| INTRODUCTION |  |  |  |
| Rationale | 3 | Describe the rationale for the review in the context of existing knowledge. | Section 1 (Introduction), paragraphs 1–2. |
| Objectives | 4 | Provide an explicit statement of the objective(s) or question(s) the review addresses. | Section 1 (Introduction), final paragraph: explicit aim statement. |
| METHODS |  |  |  |
| Eligibility criteria | 5 | Specify the inclusion and exclusion criteria for the review and how studies were grouped for the syntheses. | Section 2.2 (Eligibility criteria) — full PICOS framework reported; inclusion/exclusion criteria detailed. |
| Information sources | 6 | Specify all databases, registers, websites, organisations, reference lists and other sources searched or consulted to identify studies. Specify the date when each source was last searched or consulted. | Section 2.3 — PubMed and Cochrane Library searched up to December 2025; reference lists hand-searched. |
| Search strategy | 7 | Present the full search strategies for all databases, registers and websites, including any filters and limits used. | Section 2.3 — full PubMed search strings reported verbatim for both ACM and BrS searches. |
| Selection process | 8 | Specify the methods used to decide whether a study met the inclusion criteria, including how many reviewers screened each record and each retrieved report, whether they worked independently, and if applicable, details of automation tools used in the process. | Section 2.4 — two independent reviewers screened titles/abstracts; disagreements resolved by consensus or a third reviewer. |
| Data collection process | 9 | Specify the methods used to collect data from reports, including how many reviewers collected data from each report, whether they worked independently, any processes for obtaining or confirming data from study investigators, and if applicable, details of automation tools used in the process. | Section 2.4 — same two-reviewer process applied to data extraction. |
| Data items | 10a | List and define all outcomes for which data were sought. Specify whether all results that were compatible with each outcome domain in each study were sought (e.g. for all measures, time points, analyses), and if not, the methods used to decide which results to collect. | Section 2.2 (PICOS — Outcomes); Section 2.4 — primary outcome: prevalence of AF/AFL/AVNRT/AVRT/SVT; secondary outcomes: clinical correlates (ICD shocks, HF, thromboembolism, VA, mortality). |
| Data items | 10b | List and define all other variables for which data were sought (e.g. participant and intervention characteristics, funding sources). Describe any assumptions made about any missing or unclear information. | Section 2.4 — variables extracted: first author, year, design, sample size, age, ACM/BrS diagnostic criteria, monitoring strategy, prevalence, clinical correlates, outcomes. |
| Study risk of bias assessment | 11 | Specify the methods used to assess risk of bias in the included studies, including details of the tool(s) used, how many reviewers assessed each study and whether they worked independently, and if applicable, details of automation tools used in the process. | Section 2.6 — narrative critical appraisal at the study-set level using domains derived from the JBI checklist for prevalence studies and the Newcastle-Ottawa Scale (4 domains: representativeness, ascertainment, outcome definitions, follow-up duration). Justification for not performing item-level scoring is provided. |
| Effect measures | 12 | Specify for each outcome the effect measure(s) (e.g. risk ratio, mean difference) used in the synthesis or presentation of results. | Not applicable — no quantitative effect measure was synthesized. Outcome data presented as crude prevalence (% and absolute numbers) per study, in Tables 1 and 2. |
| Synthesis methods | 13a | Describe the processes used to decide which studies were eligible for each synthesis (e.g. tabulating the study intervention characteristics and comparing against the planned groups for each synthesis). | Section 2.5 — qualitative synthesis stratified by underlying disease (ACM vs BrS) and by SVT subtype (AF, AFL, AVNRT, AVRT). |
| Synthesis methods | 13b | Describe any methods required to prepare the data for presentation or synthesis, such as handling of missing summary statistics, or data conversions. | Section 2.5 — narrative tabulation; no statistical conversions or imputations performed. |
| Synthesis methods | 13c | Describe any methods used to tabulate or visually display results of individual studies and syntheses. | Tables 1 and 2 (study-level prevalence data); Section 3.1 (PRISMA flow Figure 1); Figures 2–3 illustrate substrate findings; Graphical Abstract. |
| Synthesis methods | 13d | Describe any methods used to synthesize results and provide a rationale for the choice(s). If meta-analysis was performed, describe the model(s), method(s) to identify the presence and extent of statistical heterogeneity, and software package(s) used. | Section 2.5 — qualitative narrative synthesis; meta-analysis not performed because of methodological heterogeneity (study designs, cohort composition, monitoring strategies) and prevalence-based outcomes. |
| Synthesis methods | 13e | Describe any methods used to explore possible causes of heterogeneity among study results (e.g. subgroup analysis, meta-regression). | Section 3.2 (Critical appraisal) — heterogeneity discussed across four domains (representativeness, ascertainment, outcomes, follow-up); subgroup discussion stratified by monitoring strategy and cohort composition. |
| Synthesis methods | 13f | Describe any sensitivity analyses conducted to assess robustness of the synthesized results. | Not applicable — no quantitative synthesis performed. |
| Reporting bias assessment | 14 | Describe any methods used to assess risk of bias due to missing results in a synthesis (arising from reporting biases). | Section 4.4 (Limitations) — publication bias toward positive associations explicitly acknowledged. No formal funnel plot performed (no quantitative meta-analysis). |
| Certainty assessment | 15 | Describe any methods used to assess certainty (or confidence) in the body of evidence for an outcome. | Section 3.2 — narrative confidence assessment based on domain-level appraisal; consistency of findings across heterogeneous designs noted as supportive of qualitative validity. |
| RESULTS |  |  |  |
| Study selection | 16a | Describe the results of the search and selection process, from the number of records identified in the search to the number of studies included in the review, ideally using a flow diagram. | Section 3.1 — full PRISMA flow described in text; Figure 1 (PRISMA 2020 flow diagram). |
| Study selection | 16b | Cite studies that might appear to meet the inclusion criteria, but which were excluded, and explain why they were excluded. | Section 3.1 — exclusion categories reported quantitatively (197 outside scope; 128 no SVT data; 37 reviews/editorials; 91 case reports; 3 non-human). |
| Study characteristics | 17 | Cite each included study and present its characteristics. | Tables 1 (BrS, 12 studies) and 2 (ACM, 15 studies) — design-level and outcome-level characteristics for all 30 included studies. Discussed individually in Sections 3.3–3.7. |
| Risk of bias in studies | 18 | Present assessments of risk of bias for each included study. | Section 3.2 — domain-level critical appraisal at the study-set level; selected studies cited as exemplars within each domain (e.g., Sacher et al. for proband-based ICD bias; BruLoop study for continuous-monitoring strength). |
| Results of individual studies | 19 | For all outcomes, present, for each study: (a) summary statistics for each group (where appropriate) and (b) an effect estimate and its precision (e.g. confidence/credible interval), ideally using structured tables or plots. | Tables 1 and 2 — per-study prevalence (% and n) of SVT, AF, AFL, and other SVT, plus mean age. Effect estimates from individual studies (HRs, ORs) cited in narrative subsections 3.5 (AF) and 3.6 (AFL). |
| Results of syntheses | 20a | For each synthesis, briefly summarise the characteristics and risk of bias among contributing studies. | Section 3.2 (Critical appraisal); Sections 3.3–3.7 begin with overview of contributing studies before synthesizing findings. |
| Results of syntheses | 20b | Present results of all statistical syntheses conducted. If meta-analysis was done, present for each the summary estimate and its precision (e.g. confidence/credible interval) and measures of statistical heterogeneity. If comparing groups, describe the direction of the effect. | Not applicable — qualitative synthesis only. |
| Results of syntheses | 20c | Present results of all investigations of possible causes of heterogeneity among study results. | Sections 3.3–3.7 — heterogeneity in reported prevalence is consistently linked to monitoring strategy and cohort composition (e.g., higher AF prevalence in ICD/ILR cohorts vs routine ECG/Holter cohorts). |
| Results of syntheses | 20d | Present results of all sensitivity analyses conducted to assess the robustness of the synthesized results. | Not applicable. |
| Reporting biases | 21 | Present assessments of risk of bias due to missing results (arising from reporting biases) for each synthesis assessed. | Section 4.4 — addressed narratively (publication bias, selective outcome reporting). |
| Certainty of evidence | 22 | Present assessments of certainty (or confidence) in the body of evidence for each outcome assessed. | Section 3.2 — narrative confidence based on domain-level appraisal and convergence of findings across heterogeneous designs. |
| DISCUSSION |  |  |  |
| Discussion | 23a | Provide a general interpretation of the results in the context of other evidence. | Section 4.1 (Summary of main findings); Section 4.2 (Pathophysiological convergence — "connexome" framework). |
| Discussion | 23b | Discuss any limitations of the evidence included in the review. | Section 4.4 (Limitations) — heterogeneity in study designs, cohort composition, monitoring strategies, diagnostic criteria across the study period. |
| Discussion | 23c | Discuss any limitations of the review processes used. | Section 4.4 — exclusion of case reports/case series, lack of formal item-level risk-of-bias scoring, no PROSPERO registration, no language restriction limitations. |
| Discussion | 23d | Discuss implications of the results for practice, policy, and future research. | Section 4.3 (Clinical implications); Section 4.5 (Conclusions and future directions). |
| OTHER INFORMATION |  |  |  |
| Registration and protocol | 24a | Provide registration information for the review, including the register name and registration number, or state that the review was not registered. | Section 2.1 — explicitly stated: not prospectively registered in PROSPERO. |
| Registration and protocol | 24b | Indicate where the review protocol can be accessed, or state that a protocol was not prepared. | Section 2.1 — protocol developed a priori by investigators; not publicly accessible. Available from corresponding author on reasonable request. |
| Registration and protocol | 24c | Describe and explain any amendments to information provided at registration or in the protocol. | Not applicable — no registration; no amendments. |
| Support | 25 | Describe sources of financial or non-financial support for the review, and the role of the funders or sponsors in the review. | Funding section — "This research received no specific grant from any funding agency in the public, commercial, or not-for-profit sectors." |
| Competing interests | 26 | Declare any competing interests of review authors. | Conflict of Interest section — declared none. |
| Availability of data, code and other materials | 27 | Report which of the following are publicly available and where they can be found: template data collection forms; data extracted from included studies; data used for all analyses; analytic code; any other materials used in the review. | Data Availability Statement — all data analyzed are available within the cited published studies; no new datasets generated. Data extraction forms and the search log are available from the corresponding author on reasonable request. |
